# Impact of Pocket Insulin Dosing Guide on Utilization of Basal/Bolus Insulin by Internal Medicine Resident Physicians

**DOI:** 10.7759/cureus.2879

**Published:** 2018-06-26

**Authors:** Chaitanya K Mamillapalli, Edward Rico, Deepika Nallala, Owaise Mansuri, Michael G Jakoby

**Affiliations:** 1 Endocrinology, Springfield Clinic, Springfield, USA; 2 Internal Medicine/Endocrinology, Paris Community Hospital, Paris, USA; 3 Internal Medicine/Endocrinology, East Tennesse State University, Johnson City, USA; 4 Internal Medicine/Endocrinology, Carle Physician Group, Urbana, USA; 5 Internal Medicine/Endocrinology, SIU School of Medicine, Carbondale, USA

**Keywords:** diabetes mellitus, insulin dosing guide, basal/bolus insulin, resident physician, hospital

## Abstract

Introduction

Basal/bolus insulin (BBI) is superior to sliding scale insulin (SSI) for diabetic patients admitted to hospital general medicine and surgery services, but little has been published on strategies to promote the utilization of BBI by resident physicians. New approaches that promote the effective management of hyperglycemia in hospitals need to be developed.

Materials and methods

A prospective study with historical controls was conducted to evaluate the impact of a pocket insulin dosing guide on the diabetes management practices of internal medicine resident physicians at the Southern Illinois University (SIU) School of Medicine, rotating on general medicine. The primary endpoint was the proportion of patients with preexisting diabetes mellitus managed with BBI. Pocket insulin dosing guides with instructions for initiating BBI and daily insulin adjustments were provided to all internal medicine residents in November 2010. BBI utilization rates were monitored over the period November 2010-February 2011 and were compared to the corresponding four-month period over the previous academic year (November 2009-February 2010), which was before the pocket insulin dosing guides were introduced (pilot study). Internal medicine house staff insulin ordering practices were subsequently evaluated for a 12-month period between October 2010-November 2011, with November 2009-October 2010 used as a historical control (study extension). New interns that were starting their residency training from July 2011 were provided with the pocket insulin dosing guides and given the same instructions as the previous academic year’s resident physicians.

Results

Historical controls (N = 579) and study patients (N = 584) were well matched, with the exception of the male gender (49% vs. 41%, P = 0.01) and diet-managed diabetes (10.5% vs. 6.4%, P = 0.01). During the pilot study, BBI increased from 12.8% of all resident insulin orders in November 2010 to 58.1% of all orders in February 2011 (P < 0.01 for trend). Overall, BBI as a proportion of all resident insulin orders was 35.7% during the pilot phase, which is a six-fold increase over the previous academic year (6%), and was also statistically significant (P<0.01). For the 12-month period of evaluation between November 2010 and October 2011, internal medicine residents ordered BBI for 41.9% of diabetes patients, compared to 16.7% of patients in the 12 months before the pocket insulin dosing guide was introduced (P < 0.01). Patients managed with BBI had higher blood glucose values at admission than patients managed with SSI (195 ± 95 mg/dL vs. 178 ± 83 mg/dL, P < 0.01) and experienced a 41 mg/dL improvement in mean daily capillary blood glucose (CBG) as compared to no change for patients managed with SSI (P = 0.01 for trend). The rate of hypoglycemia, defined as CBG < 70 mg/dL, was 2.4% for both BBI and SSI managed patients (P = 0.93).

Conclusion

The SIU pocket insulin dosing guide significantly increased the utilization of BBI, decreased SSI orders, and improved hospital glycemic control for patients with diabetes mellitus. However, over half of the general medicine patients were still managed with SSI despite the pocket insulin dosing guides. Conversion of the insulin dosing guide to a smartphone app might improve utilization of the protocol and further increase the use of BBI for inpatient diabetes management by internal medicine house staff.

## Introduction

The prevalence of diabetes mellitus among patients admitted to a hospital is significantly higher than in the ambulatory setting, and glycemic control for patients with diabetes has an impact on clinical outcomes. Using multiple data sources and including both type-1 and type-2 diabetes mellitus, the Centers for Disease Control (CDC) estimated that diabetes prevalence in the United States was at 9.4% in 2015 [1]. However, the prevalence of diabetes in the hospital setting is reported to be 17-26%, with another 12-31% of patients demonstrating other degrees of carbohydrate intolerance [2-3]. Pre-existing diabetes mellitus and newly-recognized inpatient hyperglycemia [2], as well as hospital glycemic control [4], have been demonstrated to have a measurable impact on hospital length of stay and disposition. Over 40% of healthcare spending on diabetes management is also in the hospital setting [5].

Multiple studies have demonstrated that the basal/bolus insulin (BBI) approach to the management of patients with type-2 diabetes on non-critical care services can significantly improve blood glucose control when compared to typical management approaches [6-10], improves clinical outcomes after surgery [9], and may decrease hospital length of stay [8]. Unfortunately, BBI for management of diabetes mellitus in hospitals remains underutilized. Prior to the implementation of a BBI protocol at the Carle Foundation Hospital (Urbana, IL), only 2% of diabetes patients admitted to the internal medicine service were managed with BBI [8]. Even when the BBI protocols were available, the implementation of BBI for glycemic management was limited. The mode of diabetes management and blood glucose control were monitored by a glycemic control committee at the Memorial Medical Center (MMC), a Southern Illinois University (SIU) School of Medicine teaching affiliate, and only 31% of eligible patients were managed with BBI despite a readily available protocol (Jennifer Bond, MS, RN, Director of Professional Nursing, personal communication). Hospitalist physicians at Carle Hospital used a BBI protocol for only 50% of patients, compared to a 96.7% utilization by a diabetes nurse practitioner [11].

Uncertainty regarding the initiation and adjustment of prandial and basal insulin, coupled with the tradition of managing diabetes in a hospital with sliding scale insulin (SSI), is a barrier to the utilization of BBI for inpatient glycemic management. This study evaluated the impact of a pocket insulin dosing guide on the insulin ordering practices of resident physicians who provide inpatient care on the SIU house staff covered medicine service at MMC.

## Materials and methods

A prospective cohort study with historical controls was performed. The study protocol was approved as exempted research by the Springfield Committee for Research Involving Human Subjects (SCRIHS), the institutional review board of the SIU School of Medicine.

**Figure 1:**
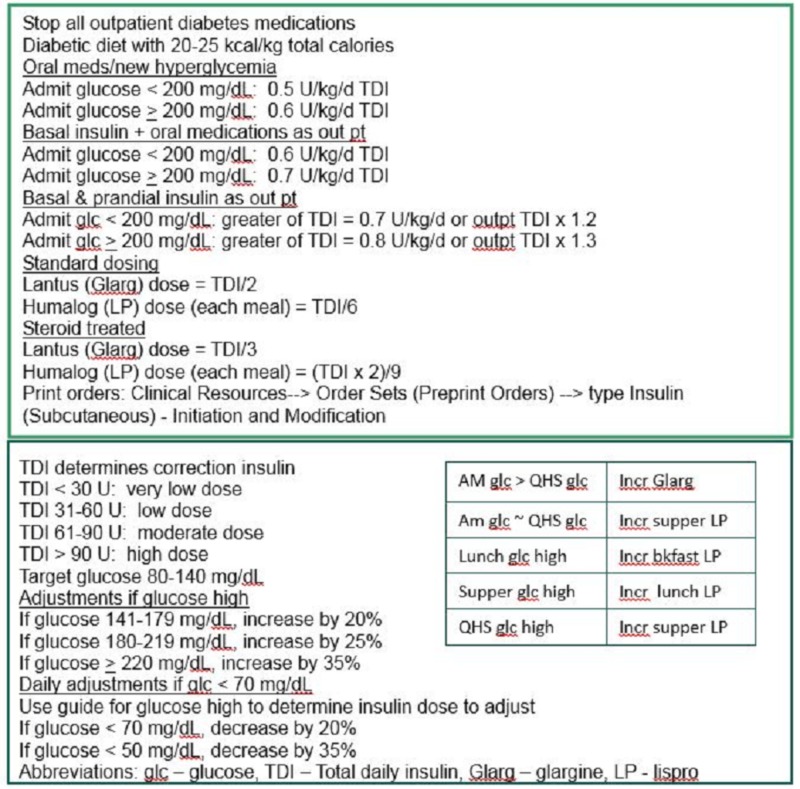
Pocket insulin dosing guide The top panel is page one, and the bottom panel is page two. Cards were printed front to back.

All SIU resident physicians were provided with a pocket guide to basal/bolus insulin dosing (Figure 1). An explanation of how to use the guide and a discussion of the rationale for BBI were presented at a resident noon conference in November 2010 by the study senior investigator (MJ). During the pilot phase of the study, the utilization of the MMC BBI protocol and capillary blood glucose readings were reviewed over the period November 2010-February 2011. Data of patients admitted between November 2009 and February 2010 were compiled as pilot phase historical controls. Due to the positive results of the four-month pilot study, data collection was extended for an additional eight consecutive months to complete a one-year study. The interval November 2010-October 2011 was the complete 12-month study period, and the interval November 2009-October 2010 was the historical control window. New internal medicine house staff for the 2011-2012 academic year received a pocket insulin dosing guide and heard the same presentation on basal/bolus insulin from the same study investigator (MJ) in July 2011. A supply of pocket insulin dosing guides was maintained by the SIU Division of Endocrinology so that resident physicians could replace lost guides as necessary.

Patient charts were reviewed to record the mode of hospital diabetes management, capillary blood glucose measurements, hospital length of stay, age, gender, type of diabetes, hemoglobin A1c (HbA1c) obtained prior to or during admission, and pre-hospital diabetes treatment. A sample size calculation for the pilot study determined that a total of 200 patients (100 in the pocket insulin dosing guide arm and 100 historical controls) were required to detect a 15% absolute increase in the utilization of the MMC basal/bolus insulin orders set and a 20 mg/dL improvement in mean capillary blood glucose (CBG) at beta = 0.8 and alpha = 0.05.

The primary study endpoint for both the pilot (four-month) and complete (12-month) studies was the proportion of patients with type-2 diabetes mellitus admitted to the SIU house staff covered general medicine service managed with basal/bolus insulin after the introduction of the pocket insulin dosing guide. Key secondary endpoints were the mean CBG for each study group, change in CBG by day of hospital admission, categorical distribution of CBG values (< 70 mg/dL, 70-140 mg/dL, and > 140 mg/dL), and hospital length of stay (LOS). Continuous variables were compared using parametric or non-parametric t-tests depending on whether the variable was normally or non-normally distributed, respectively, and categorical variables were compared using Fischer’s exact test. CBG time course curves were fit by non-linear regressions and compared by the F-test. A statistical analysis was performed using GraphPad Prism Version 5 (La Jolla, CA). Results are reported as means ± standard deviations unless indicated otherwise.

## Results

Patient characteristics for the entire 12-month study are presented in Table 1. There were 579 historical control patients and 584 pocket insulin dosing guide patients for a total of 1,163 patients in the study. The controls and intervention patients were well matched with the exceptions of higher proportions of males and patients with diet-managed type-2 diabetes in the control group compared to the intervention group (P = 0.01 for both comparisons). It is unclear if the modest gender discrepancy between the groups or the larger proportion of diet-managed patients in the control group influenced study outcomes. 

**Table 1:** Patient characteristics N - number; HbA1c - haemoglobin A1c

	Controls	Study Patients	P-value
N	579	584	
Age (years)	65 ± 14	65 ± 14	1.0
Gender (% male)	49	41	0.01
HbA1c (%)	7.7 ± 2.0	7.8 ± 2.1	0.59
Outpatient diabetes management			
Insulin (%)	40.2	44.4	0.17
Insulin + oral medication (%)	12.9	13.1	0.93
Oral medication only (%)	32.4	33.4	0.75
Diet only (%)	10.5	6.4	0.01
Unknown (%)	4.0	2.8	0.32

**Figure 2:**
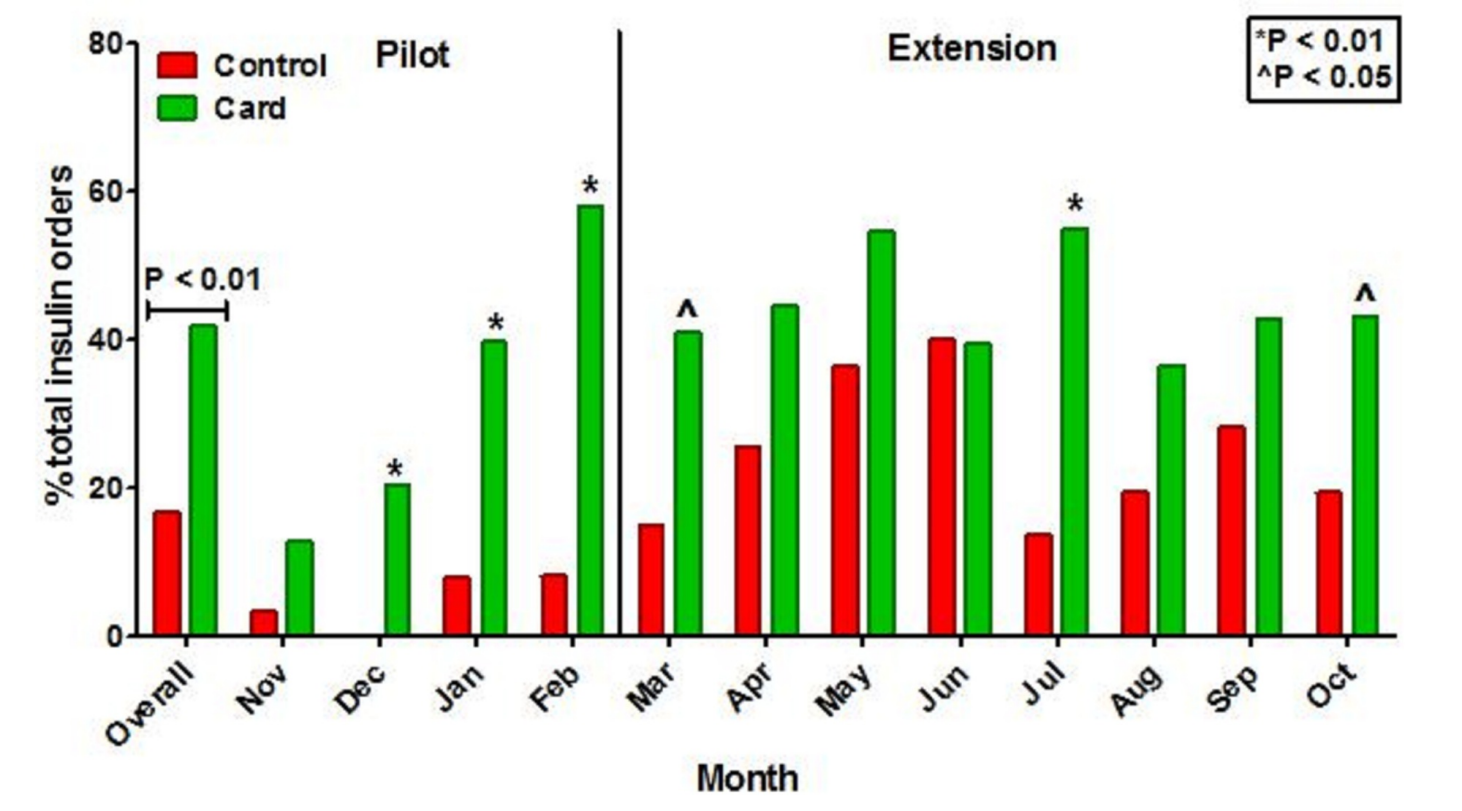
Utilization of basal/bolus insulin orders by study period Control data (red bars) are for the period November 2009-October 2010, and pocket insulin dosing guide data (green bars) are for the period November 2010-October 2011. Pocket insulin dosing guides were initially distributed at the start of the pilot phase of the study (November 2010) and again to new resident physicians at the beginning of the 2011-2012 academic year (July 2011).

Figure 2 shows the impact of the insulin pocket dosing guide on the utilization of basal/bolus insulin for management of type-2 diabetes.  During the pilot phase of the study, there was a steady increase in basal/bolus insulin for the management of type-2 diabetes, peaking at 58.1% of insulin orders in February 2011. During the pilot phase, the overall use of basal/bolus insulin increased from 6.0% during the previous year to 35.7% (P < 0.01). The glycemic control was also improved for patients managed with BBI instead of sliding scale insulin (SSI). The mean CBG for hospital days two to six was 161 ± 67 mg/dL for BBI managed patients compared to 176 ± 73 mg/dL for SSI managed patients (P < 0.01), and the proportion of CBGs in the range of 70-140 mg/dL increased from 35.9% to 42.8% (P < 0.01) for BBI managed patients with no increase in the proportion of CBGs < 70 mg/dL (2.4% vs. 2.8% SSI vs. BBI, P = 0.73).

The success of the pilot study prompted an eight-month extension for a total of 12 months of pocket insulin dosing guide evaluation. The utilization of BBI was generally higher in the historical control period extension group compared to the pilot phase of the study, but the overall use of BBI during the eight-month extension was significantly higher during the pocket insulin dosing guide intervention (24.7% vs. 44.7% control vs. intervention, P < 0.01). For the entire 12-month evaluation period, BBI utilization for type-2 diabetes management was 16.7% during the control period and 41.9% after the introduction of the pocket insulin dosing guide (P < 0.01).

**Figure 3:**
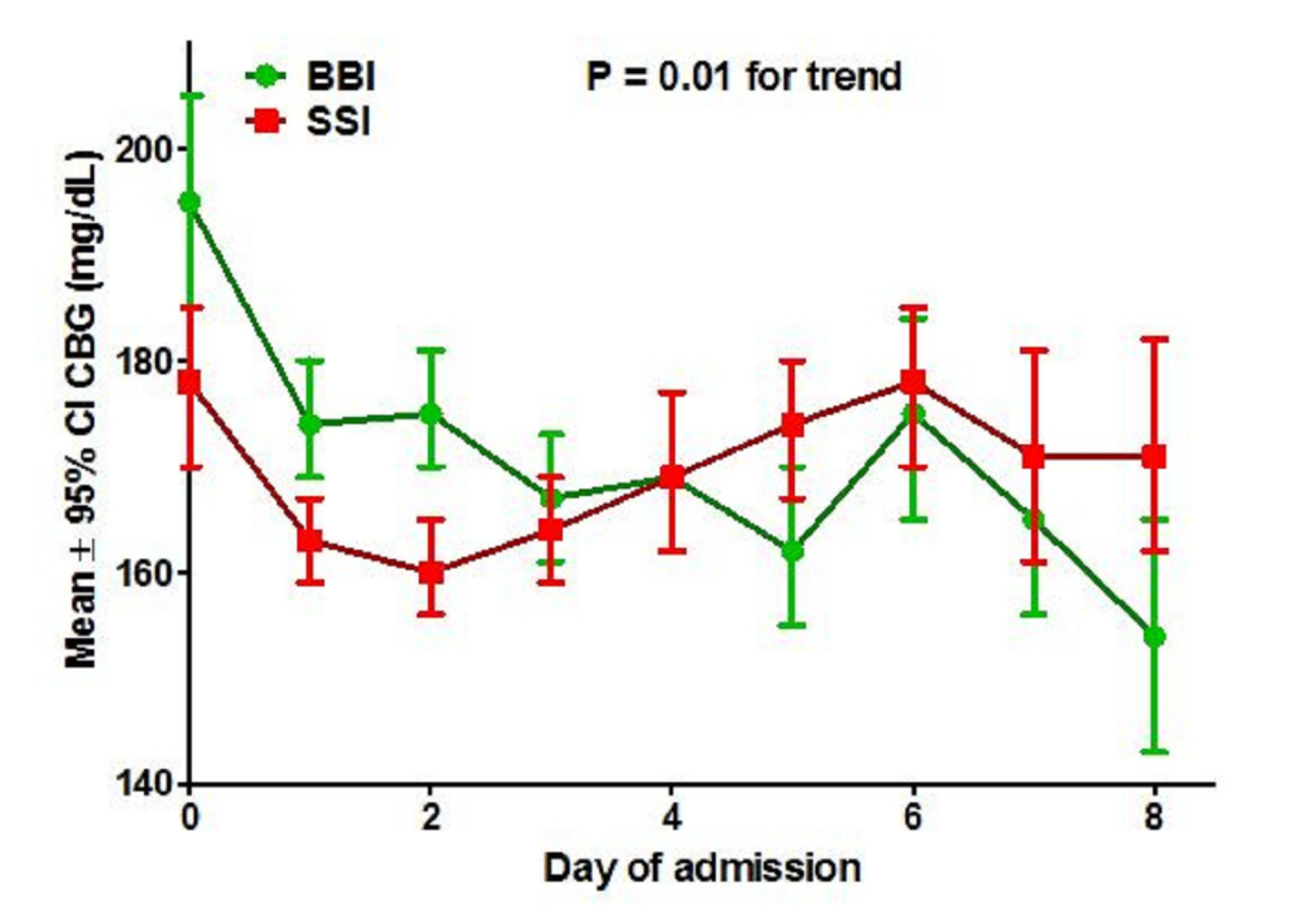
Glycemic control during the course of admission Mean ± 95% confidence interval (CI) for capillary blood glucose (CBG) in each treatment group is plotted by day of hospital admission. Admission CBG was significantly higher for basal/bolus insulin (BBI, green curve) managed patients than sliding scale insulin (SSI, red curve) managed patients, and CBG improved significantly for BBI managed patients (change in CBG – 41 mg/dL) compared to SSI managed patients (change in CBG – 7 mg/dL). Comparison of the slopes of the CBG time course curves was significant and favored BBI (P = 0.01).

The impact of BBI on glycemic control for the entire 12-month study period is presented in Table 2 and Figure 3. There were no significant differences in the mean CBG or distribution of CBGs by category for the BBI and SSI managed patients for the complete evaluation period. However, patients managed with BBI had higher blood glucose values at admission than patients managed with SSI (195 ± 95 mg/dL vs. 178 ± 83 mg/dL, P < 0.01), prompting a time course analysis to determine if glycemic control in the two groups diverged as a function of time. Curves for the mean CBG as a function of day of admission (Figure 3) showed significant improvement in glycemic control over time for BBI-managed patients (change in CBG = - 41 mg/dL) compared to almost no change in CBG from admission for SSI-managed patients (change in CBG = - 7 mg/dL). The glucose trend with time was statistically significant (P = 0.01) and favored BBI management.

**Table 2:** Measures of glycemic control over 12 months BBI - Basal/bolus insulin; SSI: sliding scale insulin; CBG - capillary blood glucose; SD - standard deviation

Parameter	BBI	SSI	P-value
Total number of CBG measurements	1675	2248	
Mean ±SD (mg/dL)	167±70	169±68	0.18
% < 70 mg/dL	2.4	2.4	0.93
% 70-140 mg/dL	39.3	37.1	0.18
% > 140 mg/dL	58.3	60.5	0.18

 

The pocket insulin dosing guide did not have a significant impact on hospital LOS. During the pilot phase, there was a trend toward shorter LOS for BBI managed patients compared to SSI managed patients (4.8 ± 5.3 vs. 5.7 ± 5.6 days, P = 0.08). However, LOS for BBI and SSI managed patients over the entire 12 month study was essentially the same (5.8 ± 5.5 vs. 5.7 ± 5.3 days, P = 0.72).

## Discussion

The SIU Division of Endocrinology pocket insulin dosing guide made a significant impact on resident physician insulin ordering practices and glycemic control for patients with type-2 diabetes mellitus on the general medicine service. Overall, BBI orders increased by 2.5-fold, and blood glucose control steadily improved during the course of admission for BBI-managed patients compared to no change in mean daily CBG for SSI-managed patients. The superiority of BBI to SSI for management of diabetes on non-critical care hospital services makes the change in resident physician diabetes management practices clinically desirable, particularly given the high prevalence of diabetes in the hospital setting and the impact of glycemic control on hospital outcomes.

The results of this study conform to previous findings that practice guidelines generally improve both the process of patient care and clinical outcomes. In a meta-analysis of 59 practice guidelines [12], Grimshaw and Russell found that over 90% (55/59) resulted in a measurable change in clinical practice and over 80% (9/11) improved patient outcomes. However, the meta-analysis included only randomized controlled trials, and the high rates of implementation and patient improvement may have been due to the supervision of guideline implementation by trial managers. Studies of diabetes management guidelines have generally occurred in the ambulatory setting, and effects on physician practice and patient outcomes have been mixed [13]. To the best of our knowledge, this is the first study specifically evaluating the impact of an insulin dosing guideline on resident physician practice and patient outcomes in the hospital setting.

Despite the substantial increase in the use of BBI for hospital diabetes management after the introduction of the pocket insulin dosing guide, the majority of patients on the general medicine service were still managed with SSI. Several factors likely account for the persistent use of SSI for inpatient diabetes management, including the limited number of hospital admissions primarily for hyperglycemia, the perception that hyperglycemia does not impact hospital outcomes, fear of hypoglycemia, and clinical inertia from over 80 years of experience with the SSI approach to hospital diabetes management [14]. 

**Figure 4:**
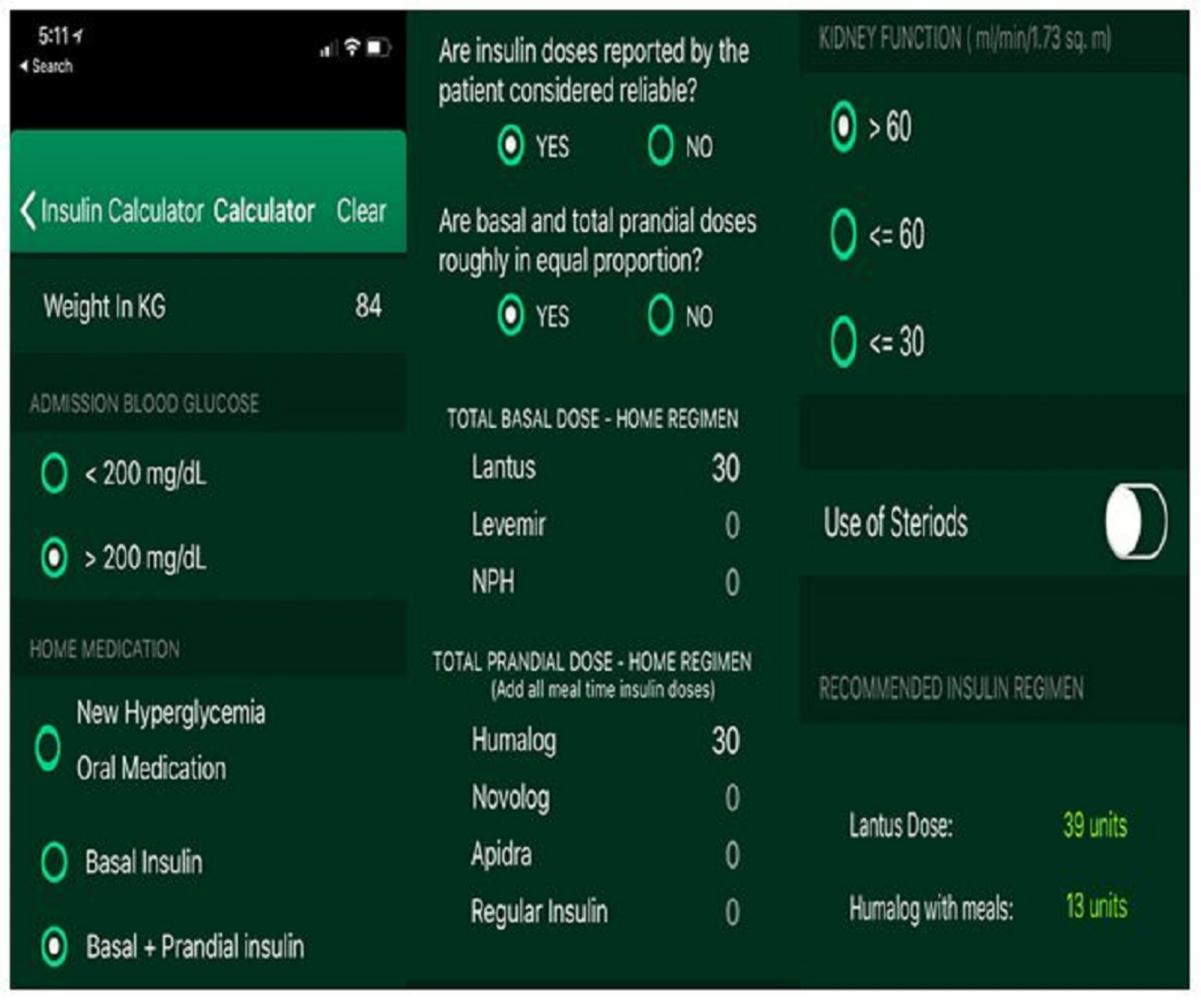
Screenshots of the SIU hospital insulin dosing calculator Each panel shows images from the hospital insulin dose calculator that has been programmed from the pocket insulin dosing guide in a representative use scenario. The insulin calculator is meant to be used to determine initial insulin doses for patients admitted to general hospital services who are ordered a regular diet and are eating reliably. “Steriods” [*sic*] is misspelled in the prototype but will be corrected in a subsequent version of the hospital insulin dose calculator.

Converting the pocket insulin dosing guide to an electronic application, including resident physicians and academic hospitalists in application development, and addressing the topic of hospital diabetes management in the residency curriculum are all strategies for building on the success of the pocket insulin dosing guide and promoting a more frequent use of BBI for inpatient diabetes management. An electronic hospital insulin dosing calculator based on the insulin pocket dosing guide has been developed (figure 4) and will undergo clinical evaluation in the near future.

## Conclusions

This study demonstrates that resident physician management of type-2 diabetes in a hospital can be changed and improved by providing a convenient pocket insulin dosing guide. Specifically, the use of BBI for diabetes management increased by over two-fold, and glycemic control improved over the course of admission for BBI-managed patients compared to no improvement for patients managed with SSI. Additional work is needed to develop tools that will promote an even higher utilization of BBI for hospital diabetes management. 
